# Randomized clinical trial: Direct‐acting antivirals as treatment for hepatitis C in people who inject drugs: Delivered in needle and syringe programs via directly observed therapy versus fortnightly collection

**DOI:** 10.1111/jvh.13701

**Published:** 2022-05-26

**Authors:** Lewis Beer, Sarah Inglis, Amy Malaguti, Christopher Byrne, Christian Sharkey, Emma Robinson, Kirsty Gillings, Andrew Radley, Adrian Hapca, Brian Stephens, John Dillon

**Affiliations:** ^1^ Tayside Clinical Trials Unit University of Dundee Dundee UK; ^2^ Tayside Drug & Alcohol Recovery Psychology Service NHS Tayside Dundee UK; ^3^ Department of Molecular and Clinical Medicine University of Dundee Dundee UK; ^4^ Ninewells Hospital and Medical School NHS Tayside Dundee Scotland; ^5^ Psychology Department NHS Fife Cupar UK; ^6^ Department of Public Health NHS Tayside Dundee UK

**Keywords:** direct‐acting antivirals, hepatitis c, needle and syringe programs, randomized controlled trial

## Abstract

Hepatitis C virus (HCV) treatment in people who inject drugs (PWID) is delivered within settings frequented by PWID, such as needle and syringe programs (NSP). The optimal direct‐acting antiviral (DAA) dispensing regimen among NSP clients is unknown. This study compared cures (Sustained virologic response 12 weeks post‐treatment, [SVR_12_]) across three dispensing schedules to establish non‐inferiority of fortnightly dispensing versus directly observed therapy. The ADVANCE HCV study was a randomized, unblinded trial, recruiting PWID attending NSP in Tayside, Scotland, between January 2018 and November 2019. HCV‐positive participants were randomized to receive DAAs via directly observed therapy, fortnightly provision or fortnightly provision with psychological intervention. A modified intention to treat analysis was used to identify differences in cures between the three treatment regimes. The study was registered with clinicaltrials.gov; NCT03236506. A total of 110 participants completed the study. 33 participants received directly observed therapy, with 90.91% SVR_12_; 37 received fortnightly provision, with 86.49% SVR_12_ and 40 received fortnightly provision and psychological intervention at treatment initiation, with 92.50% SVR_12_. Analysis showed no significant difference in SVR_12_ (*p* = 0.67). This study did not demonstrate a statistically significant difference in cure rate between groups. This provides evidence of the non‐inferiority of fortnightly dispensing of direct‐acting antivirals (DAAs) compared to directly observed therapy among PWID. It suggests that tight control of adherence through directly observed therapy dispensing of DAAs among this population offers no therapeutic advantage. Therefore, less restrictive dispensing patterns can be used, tailored to patient convenience.

**Abbreviations**
AEs: Adverse Events
BBV: blood‐borne virus
DAA: direct‐acting antiviral
HCV: Hepatitis C virus
IDU: injecting drug use
IMB: Information‐Motivation‐Behavioural
LTFU: lost to follow‐up
MDT: multi‐disciplinary team
MEM: Medical Event Monitoring
MITT: modified intention to treat
NSP: needle and syringe programs
OAT: opiod agonist therapy
pCRF: paper case report form
PWID: people who inject drugs
WHO: World Health Organization

## 1 INTRODUCTION

Hepatitis C virus (HCV) is a blood‐borne virus (BBV) spread mainly through blood‐to‐blood contact. An estimated 58 million people worldwide have chronic HCV infection, which can lead to cirrhosis and liver cancer.^1^ In 2016, the World Health Organization (WHO) published targets to facilitate elimination of HCV as a public health threat by 2030, diagnosis of 90% of individuals with chronic HCV and treatment of 80% of those diagnosed.^2^ Infection in higher‐income countries is predominantly via injecting drug use (IDU); global estimates indicate 8.5% of people infected recently injected drugs.^3^ Approximately 40% of people with recent injecting drug use are estimated to have HCV infection.^3^ This indicates that people who inject drugs (PWID) are a key population for HCV treatment, to reduce both the disease burden and infection transmission. Providing treatment pathways to PWID is a critical component of HCV elimination.

Direct‐acting antiviral (DAA) therapies are safe, efficacious and have shorter regimens than previous medications, increasing suitability for community settings.^4^ Cure, defined as elimination of virus 12 weeks post‐treatment (sustained virologic response, [SVR_12_]), was as high as 95% in clinical trials.^5^ These trials previously excluded PWID; and data showing DAA efficacy in PWID, treated in community settings, were scarce.^6^ This has since been improved, with trials taking place in community settings.^7^, ^8^ Treating PWID has proved challenging due to barriers deterring PWID from seeking treatment.^9^, ^10^ These include stigma against PWID in health settings, patients' infection risk perception and their attitudes towards illness severity and treatment.^11^ The belief that PWID will demonstrate poor adherence to treatment and have poor success rates due to illicit drug or alcohol use has hindered treatment opportunities.^12^ Many countries still restrict access to DAAs, in low‐ and middle‐income countries due to cost and fear of export to high‐income countries,^13^ and in parts of Europe, access is still restricted based on fibrosis stage or drug/alcohol use.^14^ This led to trials of DOT,^15^ which may be acceptable for people on opiod agonist therapy (OAT) who are accustomed to daily collection, but can be disruptive to those with little experience of DOT and may be a barrier to treatment.

Over the past 6 years, treatment and care for people with HCV in NHS Tayside has been scaled up and novel treatment pathways established.^16^ Together with conventional hospital‐based HCV treatment, community‐based treatment has been embedded via a combination of NHS and clinical trial‐based delivery in sites including: addiction treatment centres/community clinics, pharmacies,^17^, ^18^, ^19^ prisons and NSP.^20^ Testing and treatment are led by specialist nurses with clinician oversight and pharmacist/psychologist participation.

The use of NSP as locations for treatment has grown globally, with studies in these settings also carried out in Georgia,^21^ Australia^22^, ^23^, ^24^ and the United States.^25^

The aim of this study^26^ was to treat PWID with DAAs and determine whether provision of medication on a fortnightly basis is non‐inferior to provision via DOT, often considered the ultimate adherence aid but also restrictive for the individual.^27^, ^28^

## 2 METHODS

### 2.1 Study design

A randomized, non‐inferiority, open‐label study conducted in two NSP in Tayside, Scotland. Ethics approval by the East of Scotland Research Ethics Service (17/ES/0089; August 2017).

### 2.2 Sites

Two NSP in Dundee and Perth provided sterile injecting equipment and basic healthcare for PWID, including testing and treatment for BBVs. Signposting to other services such as substance misuse services and counselling is also provided; OAT is not provided.

### 2.3 Participants

**FIGURE 1:**
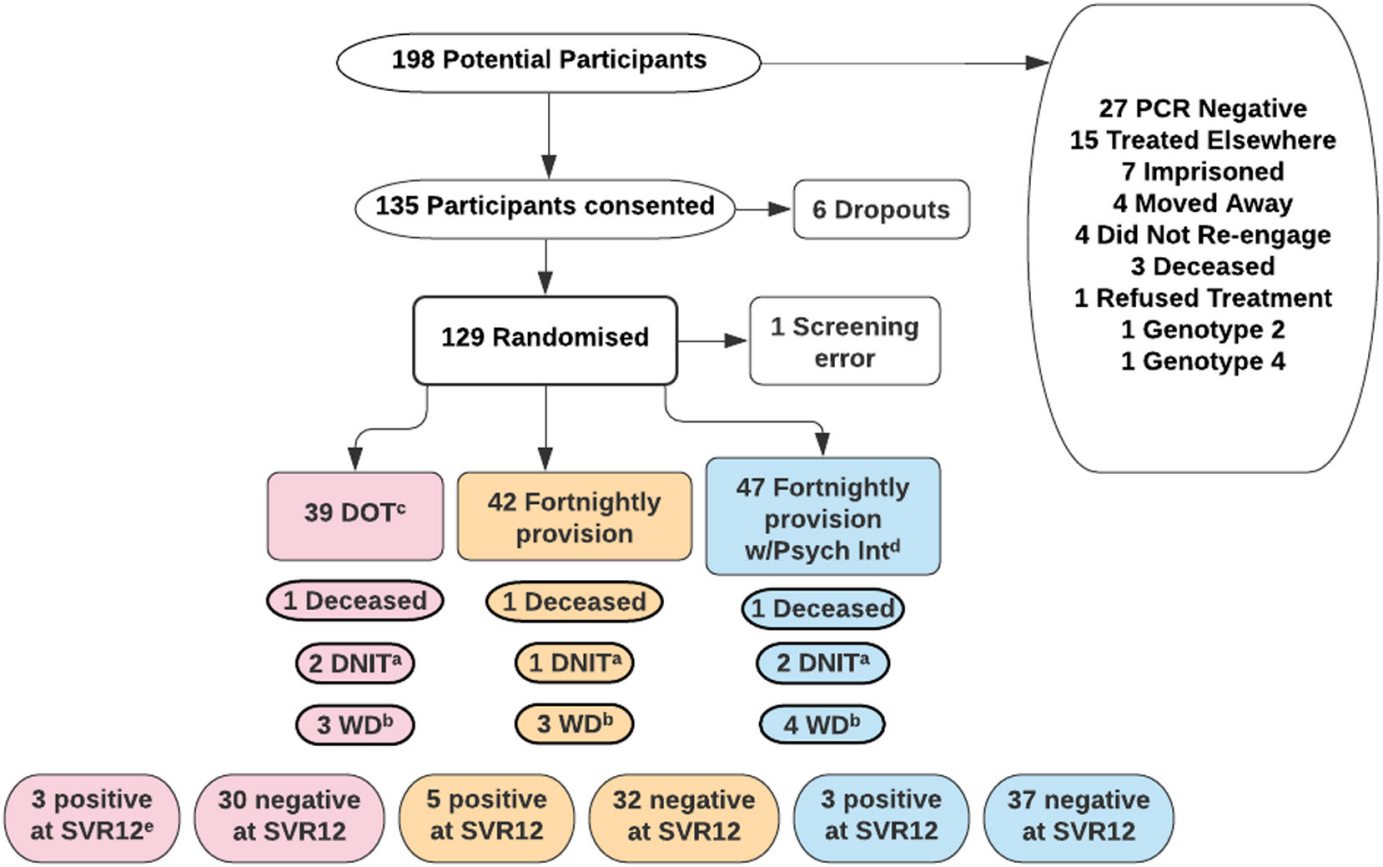
Consort diagram. ^a^DNIT, did not initiate treatment; ^b^WD, withdrawn; ^c^DOT, direct observed treatment; ^d^w/Psych Int, with psychological intervention; ^e^SVR12, test for sustained viral response at least 12 weeks post treatment

Eligibility criteria were designed to engage PWID using NSP requiring HCV treatment.^26^ Consecutive attendees at NSP with known positive HCV status were identified by specialist nurses working in NSP and given information about the study. 198 potential participants were approached, 135 consented and 129 randomized. Reasons for dropouts or non‐participation are shown in Figure 1. Participants received a fibrosis assessment using either a FibroScan® (Echosens), to determine fibrosis stage, or calculation of Fib4 from blood results. A high fibrosis score would not exclude them from the trial, but led to discussion of a multi‐disciplinary team (MDT) about safety of medication. No subjects were excluded for this reason. Written informed consent was taken, and study eligibility confirmed by a physician. Eligible participants were aged 18–70, infected with genotype 1 or 3 HCV and reported injecting drugs within 3 months of enrolling in the study. Individuals co‐infected with hepatitis B or HIV and those with severe liver disease (Childs‐Pugh B or C) were ineligible.

### 2.4 Randomization and masking

Participants were randomly assigned (1:1:1) to one of three treatment regimens using Tayside Randomisation System (TRuST), a web‐based system developed by Health Informatics Centre, University of Dundee. Treatment regimens were via DOT, fortnightly provision, or fortnightly provision with psychological intervention for treatment adherence delivered at baseline. Participants were stratified according to sex (M/F) and HCV genotype (1/3).

### 2.5 Procedures

#### 2.5.1 Treatment groups

Participants infected with genotype 1 were treated with a daily tablet containing elbasvir (50 mg) and grazoprevir (100 mg) for 12 weeks; those with genotype 3 received daily elbasvir and grazoprevir together with sofosbuvir (400 mg/day), for 8 weeks. Both regimens were prescriptions for non‐cirrhotic patients. Participants randomized to DOT were asked to attend daily and given sufficient medication to take away when the NSP were shut. Participants on fortnightly provision regimens were provided with 2 weeks' medication at a time. Participants on the fortnightly provision regimen plus psychological intervention received a one‐to‐one session, to enhance treatment adherence, delivered by a Specialist Nurse or on‐site Health Psychologist.

#### 2.5.2 Psychological intervention

The psychological intervention was based on the Information‐Motivation‐Behavioural (IMB) Skills Model of Adherence,^29^ developed to explain medication adherence behaviour in HIV treatment. It suggests that providing information about a behaviour, for example a treatment regimen, is necessary but not sufficient for such behaviour to be completed.^30^ According to the IMB model, change in adherence behaviour may be determined by providing medication and regimen information, enhancing personal and social motivation to adhere to treatment by targeting attitudes towards adherence, perceived social support for treatment adherence and subjective norms surrounding adherence of HCV treatments, in addition to developing behavioural skills associated with adherence, that is exploring capability, opportunity and self‐efficacy to perform the behaviour.^30^ Participants completed a booklet, ‘Hepatitis C and Me’, which contained general and personalized information on HCV medication. It also contained exercises designed to explore and enhance personal and social motivation for adherence and a behavioural action plan to facilitate adherence to DAAs, for example exploring barriers and facilitators to adherence to DAA treatment. This can help develop coping strategies and reflect on self‐efficacy to enact strategies. The intervention was a single session of up to 1 h, delivered prior to the first medication dose. The intervention followed the principles of node‐link mapping to facilitate engagement around adherence.^31^ This was different from HIV trials where interventions based on the model were cognitive‐behavioural in nature and longer.^32^

#### 2.5.3 Assessments

Baseline assessment included blood tests to confirm eligibility, demographic information, social history and information about drug habits which were recorded in the paper case report form (pCRF).^26^ Blood tests and information about illicit drug‐taking were collected at the end‐of‐treatment visit. At the final study visit (SVR_12_), blood was taken and analysed by the health board laboratory to test for active HCV infection to confirm cure. The final study visit could be up to 12 weeks from the scheduled SVR_12_ date allowing maximum opportunity to be seen and to avoid classifying individuals as lost to follow‐up (LTFU) prematurely. Participants having an SVR_12_ test outside this window were included but classified as ‘late’. This allowed capture of all participants who initiated treatment and had a test post‐treatment. No participants were re‐treated.

At all visits, compliance with medication regimen was assessed. Attendance of participants following DOT was recorded. Participants on fortnightly regimens returned any remaining medication when they attended the NSP for their next batch of medication. Participants infected with genotype 3 HCV were given sofosbuvir in bottles fitted with Medical Event Monitoring (MEM) caps (Westrock Switzerland, MEMS8 TrackCap 38 mm CR). Participants missing more than seven consecutive doses were withdrawn from the study.

#### 2.5.4 Data collection

Data collected at each visit were entered into a pCRF and subsequently an electronic CRF (eCRF) using Openclinica open source software V3.1.3.1 (https://openclinica.com/). Data were stored in an anonymized state identified by study number. No personal information was shared beyond the clinical care and research team.

### 2.6 Outcomes

The primary outcome was the evidence of a non‐inferior rate of cure in participants treated by fortnightly provision of medication and fortnightly provision with psychological intervention when compared to those treated by DOT. The success or failure to obtain a cure would be an indicator of suitable adherence to the treatment regimen.

Safety and Adverse Events (AEs): Due to the high level of co‐morbidities in this cohort a number of adverse events that would normally be reported as SAEs were defined in the protocol as being excluded from reporting requirements. They were recorded on participants' AE logs as non‐reportable SAEs.^26^

### 2.7 Statistical analysis

Analyses were conducted according to modified intention to treat (MITT); all participants who had at least one dose of therapy were included. Analysis complied with ICH E9 ‘Statistical Principles for Clinical Trials’ and was performed by the UKCRC*‐*registered Tayside Clinical Trials Unit. All participants that had an SVR_12_ outcome (either negative or positive) are included. The primary outcome of SVR_12_ was assessed using logistic regression modelling. We defined the fortnightly therapies as being non‐inferior to the DOT treatments if the cure rates were no more than 14% lower than DOT rates. Previous studies have shown that cost effectiveness of therapy is maintained with a non‐inferiority limit of 14%.^33^, ^34^ A 95% SVR rate was assumed (based on published studies)^5^ in the DOT arm of the trial in this population. At a 5% significance level and 90% power, the study would need a sample size of 42 in each group 126 in total. To allow for dropouts, the aim was to recruit 135 individuals, 45 per group. This low estimated drop‐out rate was justified due to the established regular contact the participants had with the NSP to collect injecting equipment and maintain contact.

Analysis was performed using SAS version 9.4.

The study was not overseen by a data monitoring committee since the safety risk to participants was assessed to be very low. Data integrity was assessed by a study management group, and the study was subject to external monitoring visits every 6 months. The study is registered on Clinicaltrials.gov, number NCT03236506.

### 2.8 Role of the funding source

The study funder had no role in study design, data collection, data analysis or data interpretation. They reviewed this manuscript but did not make substantial changes to the text. The corresponding author had access to all data in the study and had final responsibility for the decision to submit for publication.

## 3 RESULTS

129 participants were randomized between January 2018 and November 2019 (1 was removed due to a screening error). 96 completed the study by attending for an SVR blood test within 24 weeks from end of treatment. 14 obtained their primary outcome later than 24 weeks after end of treatment.

Three deaths were reported during the study, two were after the participants' treatment period and unrelated to the study drugs. One participant died before commencing treatment. All deaths were attributable to recreational drug overdose. Ten participants withdrew by choice. Two participants were held in custody in prisons outside the health board. One participant was withdrawn due to an SAE and two participants withdrawn for other reasons.

Baseline demographics of age, gender, living situation, source of income, alcohol consumption and genotype are shown in Table 1. In all three groups, most participants were male, a mean age of 36.5 years with HCV genotype 3. Most lived in rented or owned accommodation with one third being homeless. Most received government benefits; some supplemented this with other income sources. 79.78% of participants reported drinking no alcohol.

**TABLE 1:** Characteristics of study population

Characteristic	DOT (n = 39)	Fortnightly provision (n = 42)	Fortnightly provision with psychological intervention (n = 47)
Female (%)	10 (25.64)	11 (26.19)	15 (31.19)
Age, mean (SD)	36.2 (8.20)	35.7 (7.33)	37.7 (7.53)
Genotype 1 (%)	15 (38.46)	19 (45.24)	18 (38.30)
Living situation			
Owned/rented (%)	24 (61.53)	24 (57.14)	31 (65.96)
Supported accommodation (drug related) (%)	1 (2.56)	3 (7.14)	2 (4.26)
Homeless (%)	14 (35.89)	15 (35.71)	14 (29.79)
Source of income			
Unemployed, on government benefit (%)	20 (51.28)	23 (54.76)	30 (63.83)
Unemployed, on government benefit & at least one other source of income (%)	18 (46.15)	15 (35.71)	14 (29.79)
Casual work and government benefit (%)	1 (2.56)	1 (2.38)	0
No government benefit but other source of income (%)	0	3 (7.14)	3 (6.38)
Alcohol consumption per week			
No alcohol consumption (%)	32 (82.05)	33 (78.57)	37 (78.72)
Between 1 and 20 units (%)	5 (12.82)	8 (19.05)	8 (17.02)
Over 20 units (%)	2 (5.13)	1 (2.38)	2 (4.26)

All participants were active PWID, having reported injecting drugs within 3 months prior to enrolment. Most participants reported injecting on at least 5 occasions in the past week (Table 2) and were not being prescribed OAT at study enrolment. The mean age at which participants first injected illicit drugs was 20.10 years. Problem drug use is defined as long term or regular injecting drug use.

**TABLE 2:** Drug injecting history

Injecting history	DOT (*n* = 39)	Fortnightly provision (*n* = 42)	Fortnightly provision with psychological intervention (*n* = 47)
Within the last week (%)	27 (69.23)	28 (66.67)	33 (70.21)
Injections within the last week Mean number (range, SD)	8.04 (0–35, 7.65)	5.68 (0–14, 2.68)	5.21 (0–10, 2.55)
Within the last month (%)	4 (10.3)	8 (16.3)	6 (12.8)
Within the last 3 months (%)	8 (20.5)	6 (14.3)	8 (17.0)
Age at first injection, Mean years (range, SD)	19.87 (7–40, 7.71)	19.31 (10–43, 6.73)	21.13 (9–43, 7.51)
Age of problem drug use, Mean years (range, SD)	23.08 (14–40, 7.22)	21.57 (14–43, 6.60)	23.28 (13–43, 7.07)
Currently prescribed OAT	15 (38.46)	12 (28.57)	13 (27.66)

**FIGURE 2:**
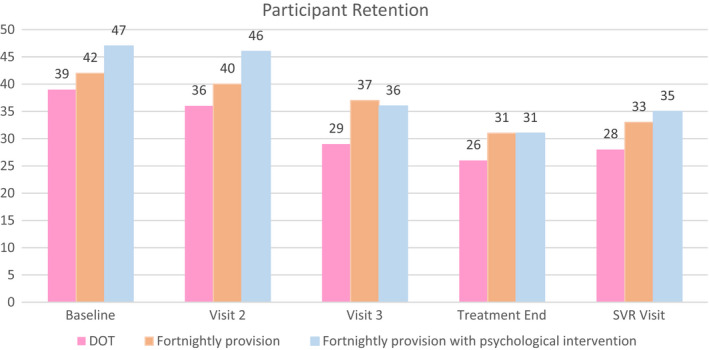
Participant retention

Figure 2 shows participant retention through the study per treatment. There was no statistically significant difference across the three dispensing regimens in the number of participants attending each visit. This implies that participants were no more likely to miss fortnightly visits to collect their treatment than those who collected daily.

The overall rate of participants testing negative for HCV at SVR_12_ was 90% (99/110; Table 3). This was not statistically different in any of the treatment groups. 96 participants completed the study per protocol with an overall per protocol SVR_12_ of 91.67%. 14 participants had an SVR test over 24 weeks from the scheduled date with a cure rate of 78.57% (11/14; Table 3).

**TABLE 3:** Cure assessed within and beyond 24 weeks

Treatment	N	SVR_12_ visit outcome	
		Positive	Negative
DOT cure test within 24 weeks	28	2 (7.14%)	26 (92.86%)
DOT cure test beyond 24 weeks	5	1 (20.00%)	4 (80.00%)
Fortnightly provision cure test within 24 weeks	33	4 (12.12%)	29 (87.88%)
Fortnightly provision cure test beyond 24 weeks	4	1 (25.00%)	3 (75.00%)
Fortnightly provision with psychological intervention cure test within 24 weeks	35	2 (5.71%)	33 (94.29%)
Fortnightly provision with psychological intervention cure test beyond 24 weeks	5	1 (20.00%)	4 (80.00%)
Overall cure test within 24 weeks	96	8 (7.27%)	88 (91.67%)
Overall cure test beyond 24 weeks	14	3 (21.43%)	11 (78.57%)
Total	110	11 (10.00%)	99 (90.00%)

90% of participants achieved a cure, 41.82% were Genotype 1 and 48.18% were Genotype 3. There was no statistical difference between genotypes. 10% of participants were HCV positive at 12 weeks post‐treatment. Analysis of SVR_12_ outcome in each group (Table 4) showed no significant difference between treatment interventions (*p*‐value = 0.67). Cures among participants with fortnightly provision of treatment, ± psychological intervention, was not inferior to DOT.

**TABLE 4:** Treatment comparison

Treatment comparison	Effect difference (confidence interval)	Odds ratio	*p*‐Value
Fortnightly provision vs. DOT	−4.86% (−20.22, 11.62)	0.64 (0.14–3.00)	0.57
Fortnightly provision vs. Fortnightly provision with psychological intervention	−6.50% (−8.82, 24.51)	0.53 (0.11–2.45)	0.41
Fortnightly provision with psychological intervention vs. DOT	1.75% (−11.88, 16.59)	1.22 (0.23–6.61)	0.82
Overall (chi‐square test)			0.67

## 4 DISCUSSION

### 4.1 Main findings

This study showed equivalence of DAA by DOT or two‐weekly dispense (± psychological intervention) with high overall SVR_12_ of 90%. This is comparable with other real‐world cohorts,^5^ especially with MITT. Per protocol SVR for the groups was 92.86% for DOT, 87.88% for fortnightly provision and 94.29% for fortnightly provision with psychological intervention. The study demonstrates delivering HCV testing, treatment and cure via NSP to actively injecting PWID is feasible. It further suggests that treatment within such settings can be provided without requirements for observation of medication consumption or delivery of interventions to improve adherence. These are simple steps that can be taken in community settings worldwide to move towards elimination of HCV. The study was purposefully designed to be as pragmatic as possible. It represents a real‐world generalizable outcome, without the biases associated with conventional clinical trial exclusions. Participants were recruited within an NSP, an easily accessible, familiar setting, removing requirement to travel to a hospital clinic. Recruitment, treatment and care were by specialist nurses, who routinely provided HCV and harm reduction care, with wider multi‐disciplinary team support as required. This can be replicated in most community settings in other high‐ and middle‐income nations.

Evidence that fortnightly provision is non‐inferior to DOT may allow healthcare services to move away from practising DOT in this, and similar, patient groups^35^ or demonstrate that new services do not need to adopt such practices. It demonstrates that active PWID who may not be on OAT can be engaged in therapy successfully without the need for daily pick‐up. The COVID‐19 pandemic has seen a shift away from daily dispensing of OAT, and this study provides evidence that this will not adversely impact on co‐treatment of HCV. Fortnightly dispensing can allow for a disadvantaged population to be treated for HCV with dignity and discretion^36^, ^37^ as well as reduce the cost of travelling. Actively involving individuals in choice of treatment is an essential component of trauma‐informed care and has been shown to improve treatment outcomes.^15^

Many of the participants who were allocated the psychological intervention engaged productively and collaboratively in this activity. However, no difference in cure rate was observed, implying that treatment adherence was sufficient in all three dispensing groups. The efficacy of DAAs is very high and accommodates the variance in treatment adherence that may occur in this group.

This study is an excellent example of delivery of micro‐elimination, the intervention focussed on a specific group of patients with a novel venue of access and need to create a specific pathway to reach them. It formed part of a wider programme between 2017 and 2020, designed to scale‐up treatment of PWID in Tayside through pragmatic clinical trials and novel treatment pathways. The programme was successful and HCV elimination, as defined by the WHO,^2^ was achieved in October 2020.^38^ This made Tayside one of the first regions in the world to attain elimination. To achieve HCV elimination or micro‐elimination^39^ or to contemplate treatment as prevention strategies,^16^ engagement and treatment of PWID who have HCV infection are vital. This project demonstrates that this is achievable and that denying treatment to PWID due to disease progression or drug use is not consistent with WHO elimination strategies.

### 4.2 Limitations of the study

Loss of communication with participants was a common feature throughout the study. However, the specialist nurses proactively sought to engage participants and remind them of study visits. A mobile phone offered greater access by enabling text messaging, although some participants changed phone number during the study.

Study treatment for male participants who were taken into custody was maintained. However, since there are no local women's prisons, females were held outside the Health Board area and their treatment and study participation was terminated. This directly impacted two female participants who were removed from the study for this reason. The study team ensured that all participants were offered follow‐up by health professionals within penal establishments, regardless if out of area, to determine the impact of their completed or incomplete treatment. This highlights an unintended inequality in female participants being able to complete their treatment.

### 4.3 Suggestions for future research

Mirroring the therapeutic advances observed in HCV therapies, OAT have recently seen novel and emerging treatment options, such as injectable prolonged release buprenorphine.^40^ For example, OAT patients on monthly injectable buprenorphine will have reduced necessity to attend their dispensing pharmacies. Future research should focus efforts to trial monthly dispensing of DAA treatments.^41^ Although not the focus of this paper, given the continued risk behaviour observed in this population, future research should investigate targeted interventions for prevention of HCV reinfection. Reinfection rates in this study are a secondary outcome and will be reported separately. The follow‐up period is 5 years.

### 4.4 Conclusion/Implications

The results of this study show that PWID can be successfully treated for HCV by Specialist Nurses within a community NSP. SVR_12_(90%) is equally high whether medication is dispensed on a fortnightly or daily basis. This provides evidence in support of the non‐inferiority of fortnightly dispensing of DAA compared to DOT among PWID. It suggests that using only DOT methods of DAA dispensing among this population offers no therapeutic advantage.

Our findings align with recent HCV treatment guidelines^42^ that suggest minimal on‐treatment monitoring is required to ensure therapeutic efficacy. Regions with a high level of PWID which drives BBV transmission may consider providing testing and treatment via local NSP.

## Data Availability

The data that support the findings of this study are available from the corresponding author upon reasonable request.
